# Seasonal Dynamics of the Leaffooted Bug *Leptoglossus zonatus* and Its Implications for Control in Almonds and Pistachios

**DOI:** 10.3390/insects10080255

**Published:** 2019-08-19

**Authors:** Kent M. Daane, Glenn Y. Yokota, Houston Wilson

**Affiliations:** 1 Department of Environmental Science, Policy, and Management, University of California Berkeley, Berkeley, CA 94720-3114, USA; 2 Department of Entomology, University of California, Riverside, Riverside, CA 92521, USA

**Keywords:** large bug damage, hemiptera, leaffooted bug, seasonal development, overwintering survival

## Abstract

*Leptoglossus zonatus* is a polyphagous pest found throughout much of the Western Hemisphere. In California, *L. zonatus* attacks almond, pistachio, pomegranate, and walnut crops, but the seasonal use of and economic damage to these crops varies. To better understand the seasonal changes of *L. zonatus* populations and to improve monitoring programs in California’s San Joaquin Valley, we caged overwintering adult males and females and then followed the resulting population dynamics over a one-year period. There were three generations over the one-year period, although eggs, nymphs, and adults overlapped among successive generations. From an initial 75 overwintering adult females, there were 1214 egg strands, 16,692 nymphs, and 4900 adults recorded during the one-year period. Depending on the generation, the number of nymphs per egg strand ranged from 11.3 to 14.3; the sex ratio was close to 1:1 with the exception of one female-biased cage; and nymph mortality ranged from 22.0% to 39.5%. Adult females isolated from each generation produced 2.4–5.1 egg strands per female that totaled 41.7–61.7 eggs per female with a 67.1–86.8% successful hatch rate. We find that the adult is the overwintering stage, as more adults (without food provisions) survived the winter compared to medium-sized or large-sized nymphs provided with both food and water. The results are discussed with respect to the development of *L. zonatus* control and monitoring programs for California’s multi-billion-dollar (US) nut crops.

## 1. Introduction

**Figure 1:**
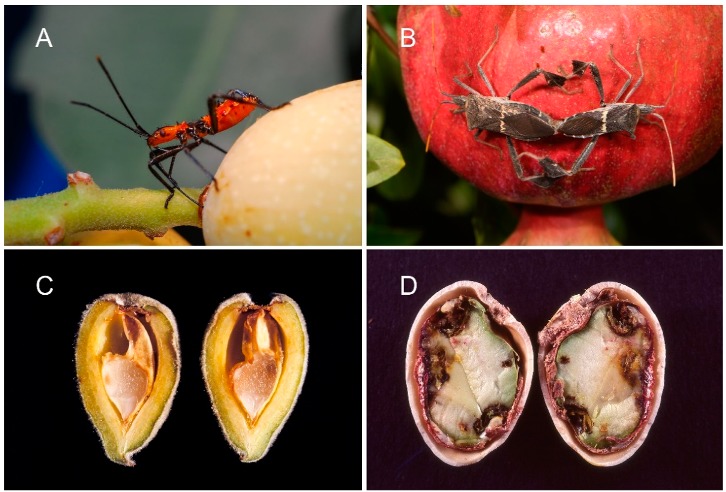
*Leptoglossus zonatus* has become one of the more important hemipteran pests in California almonds and pistachios; shown here (**A**) as an early instar feeding near the petiole of a pistachio nut, (**B**) adults mating in a pomegranate orchard where they commonly build large numbers in the fall, (**C**) feeding damage to an almond showing style puncture and kernel damage, and (**D**) feeding to a pistachio, showing sites of style entrance, kernel necrosis, and fungal contamination or stigmatomycosis (photos: K.M.D.).

California almonds and pistachios are attacked by a variety of insect pests [1,2]. While the navel orangeworm *Amyelois transitella* (Walker) is arguably the most important [3,4], hemipterans can be a damaging group that often require annual treatments [5]. Hemipteran pests of these nut crops are commonly divided into ‘small’ and ‘large’ bugs. Small bugs include several species of Miridae and Rhopalidae, most importantly *Closterotomus norvegicus* (Gmelin), *Phytocoris relativus* Knight, and *Lygus hesperus* Knight [6]. These bugs can be abundant early in the season and may cause significant crop loss through epicarp lesions (damage to the outer shell) and fruit drop but cease to cause damage after the shell begins to harden [7,8,9]. The large bugs are composed of species of Pentatomidae and Coreidae, most notably the stink bugs *Thyanta pallidovirens* (Stål), *Chlorochroa uhleri* (Stål), and *C. sayi* (Stål), and *Chinavia hilaris* Say, and the leaffooted bugs including *Leptoglossus zonatus* (Dallas) and *L. clypealis* Heidemann [5]. Large bugs can cause the same damage as their smaller counterparts during the first half of the season; however, during the latter half of the season (from shell-hardening until harvest), large bugs can continue to puncture the shell, causing kernel necrosis (damage to the nut meat) and facilitating stigmatomycosis (a mold that infests the nut) [7,10,11] and *Botryosphaeria* fungal contamination in pistachios [12] (Figure 1).

Monitoring and control of this assemblage of hemipteran pests is a primary integrated pest management (IPM) objective for California’s almond and pistachio industries. This has become increasingly important as pyrethroids have been the primary control tool for hemipterans [2,13] and their repeated use has resulted in resistance development for navel orangeworm [14]. Three factors determine the amount of bug damage: seasonal timing of feeding relative to periods of crop vulnerability, size of the insect (or, better stated, how large the insect’s mouthparts are) and, of course, pest abundance [5]. Early in the season (mid-February to March for almonds, and April to mid-May for pistachios), damaged nuts often drop from the cluster [9,10,15]. For almonds, this represents crop loss [16], whereas for pistachios, yield loss may be compensated by the natural nut drop [5,9]. After fruit load is set but before shell lignification (hardening) is complete, damaged nuts can remain in the cluster and, in pistachios for example, the resulting epicarp lesion can stain the outer shell, thus lowering market value; and feeding wounds can introduce fungal contamination (such as stigmatomycosis) [7,10,17]. Later in the season, the shell is too hard for nymphs or smaller bugs to penetrate [10] and epicarp lesions no longer rapidly form in response to insect wounds after peroxidase activity in the pericarp declines during fruit maturation and shell hardening [18].

While this seems simple enough, these three factors can result in many different scenarios that impact optimal spray timing in almonds and pistachios and makes control decisions based on a simple calendar-date spray schedule for hemipteran pests less than ideal. Moreover, because of the importance of bug size and shell hardness, the activity of adult leaffooted bugs and stink bugs early in the season presents the greatest potential for crop loss. Predicting population densities and overwintering survival is especially important for leaffooted bugs that, in some years, can move into the orchard in large numbers early in the season and cause significant crop loss [16]. Current management strategies for *Leptoglossus* are limited, in part, because of inadequate monitoring strategies [19]. Pheromones are known to play a role in the reproduction and aggregation of both *L. clypealis* and *L. zonatus* [20,21,22]. However, they are not yet commercially available for monitoring, and while some of the pheromones have been characterized and synthesized, no field applications have been developed. Kairomones and herbivore-induced plant volatiles may also play a role in leaffooted bug dispersal and the formation of aggregations, which could alone or in combination with pheromones be used as part of either an improved monitoring tool or attract-and-kill control strategy [19].

Predicting the seasonal occurrence and abundance of an insect pest are essential to the development of IPM systems, but this cannot happen without effective monitoring tools [23]. Barta [24] studied temperature development and winter survival of *Leptoglossus occidentalis* Heidemann as an invasive pest in Slovakia. These data demonstrated that overwintered adults became active in mid-March, and while populations were univoltine in southern Slovakia, *L. occidentalis* could have up to four generations per year depending on the locality in Europe. Studying *L. zonatus* as a pest of satsuma mandarin, Xiao and Fadamiro [25] detailed some of the life table parameters on satsuma fruit in relation to its field ecology and different control options. Similarly, Panizzi [26] studied *L. zonatus* on green bean pods, corn, and soybeans and showed that this pest’s developmental time and nymphal survivorship varied on the different food sources. Information is still needed for California populations of *L. zonatus* on nut crops to provide a better understanding of their seasonal abundance and overwintering biology in order to better predict winter-spring migrations into almond and pistachio crops. Here, we determined the number of annual generations per year and the overwintering survival under season-long ambient temperatures in California’s San Joaquin Valley and discuss the implications of this work for control programs in almonds and pistachios.

## 2. Materials and Methods

### 2.1. Annual Generations and Seasonal Development

The number of annual generations and seasonal biology of *L. zonatus* were investigated at ambient temperatures for California’s San Joaquin Valley. To begin, ~300 adults were collected from a 10-year-old commercial planting of ‘*cv.* Purple Heart’ pomegranates near Porterville, California on 9 November. *Leptoglossus zonatus* can form large populations on pomegranates in the fall, making collections relatively easy. The collected insects were housed in large (35 × 35 × 80 cm) organdy-screened wood cages, provisioned with green beans (replaced as needed) for food and a potted (3.8 L) cypress (*Cupressus sempervirens* L.) that increased the surface area available to the bugs and provided an additional food source. Small bamboo skewers (~3 mm diameter × 25 cm length) were used as an oviposition substrate, although the adults would occasionally oviposit on the organdy part of the cage as well. The cages were placed outdoors at ambient temperatures but protected from rain and direct sunlight at the Kearney Agricultural Research and Extension Center near Parlier, California. Insects in these holding cages were observed twice weekly, and the trial was initialized when mating was first observed in late March.

On 29 March, 25 female and 25 male *L. zonatus* from the holding cages were placed in each of three cages newly provisioned with the green beans and cypress that were refreshed as needed and located outside but protected from rain and direct sunlight. Approximately every 3–7 days, we recorded the number of new egg strands (new egg strands were determined by the increase in total egg strands on each observation period) and removed any dead adults, recording their number and sex. To estimate population abundance and nymphal development, we also removed and counted nymphs that developed to the 2nd to 4th instar, recording the developmental stages present and placing the nymphs into a new, similarly provisioned cage. In this manner we were able to keep adults from the overwintering generation (F_0_) separate from adults of the first summer generation (F_1_). On each collection, we attempted to relocate 60–90% of the nymphs, thereby providing a count of the relative abundance of new nymphs.

Each series of three cages (A, B, or C) followed the same lineage, with nymphs from cage A0 being moved to cage A1, then to A2, and so. In the new cages with F_1_ nymphs (A1, B1, and C1), we similarly recorded the number of adults and egg strands; when adults died, they were removed from the cage as before, recording their numbers and gender. This process was repeated for a full year, so as nymphs in the F_1_ cages developed to the 2nd to 4th instar, they were removed from these cages and placed into the respective F_2_ cages and later the F_3_ cages.

#### Egg Strand Production and Egg Development

To better determine adult reproductive potential, overwintered adult females (n = 20) and newly formed adult females from the first (n = 60) and second (n = 10) generations were individually isolated in 40 × 85 mm Petri dishes and held, similar to the generation study, at ambient temperatures in the San Joaquin Valley. Petri dishes were ventilated with two 20 mm organdy-covered holes on the lid, and adults were provisioned with green beans and peanuts. After 2–3 days, males were added to each container for a 3–4-day period and then removed, and then added again every 14 days. Every 2–5 days thereafter, the containers were checked for new egg strands, which were then isolated and held to determine the number of eggs per female, the number of 1st instars that successfully hatched, and the egg development time for each generation.

### 2.2. Overwintering Survival of Nymphs and Adults

To determine the effect of development stage and food provisioning on overwintering survival, *L. zonatus* were collected from two San Joaquin Valley locations: a ~10-year-old ‘hedgerow’ planting of ‘*cv.* Wonderful’ pomegranates near Reedley, California, and a ~10-year-old commercial planting of ‘*cv.* Purple Heart’ pomegranates near Porterville, California. About 900 *L. zonatus* adults and nymphs were collected in mid-November and placed in the organdy-screened holding cages described previously (Section 2.1). The trial began on 13 December when 300 medium-sized nymphs (2nd–3rd instar), 300 large-sized nymphs (4th–5th instar), and 300 adults were collected and each divided into six groups of 50 individuals. Insects from each size category were then randomly assigned to food or no food treatments, with the food treatment containing green beans, peanuts, and water, and the no food (control) treatment containing water only (food and water provisions were changed weekly). For each size category and food provisioning combination, the tested insects were divided into five large (20 × 85 mm) ventilated Petri dishes (10 insects per dish). The dishes were assigned to three organdy-screened cages placed outdoors as described above. The Petri dishes were checked at 1–2-week intervals and each insect’s condition (alive or dead) was recorded. The trial was discontinued after 104 days (26 March) when there was complete mortality in the nymph treatments and, concurrently, mating was observed in the adult treatments. At that time, adults from both food and no food treatments were provided fresh food and held for two additional weeks to record egg production.

### 2.3. Data Analysis

Results are presented as sample means ± SEM. The number of seasonal generations is graphically presented. The number of nymphs hatching per egg strand was estimated by the total number of nymphs per total egg strands per cage; nymph mortality, from 2nd–4th instar, was estimated by the number of nymphs added to each cage minus the number of adults later removed; offspring sex ratio was determined when the dead adults were removed from each cage. For individual adult female egg production and longevity, only individuals that produced at least one egg strand were included in the data analysis.

In the overwintering survival trial, treatments were compared using ANOVA with development stage as the factor and food provisioning as the covariant. Longevity was further separated for each stage-food combination using Tukey’s HSD test. Survival data were analyzed with the Cox survival analysis model and Kaplan–Meier estimator. Survival among the treatment combinations was compared using the parametric model function, with age category as the strata and food as the covariate; the output provided the Mantel–Haenszel log-rank test for strata separation (chi-square) and a *Z*-test for impact of the covariate. We additionally used the nonparametric model function for all possible pair-wise comparisons (food and development stage); in these analyses, the experiment-wide error rate used was α = α/n, where α = 0.05 and n is the number of possible pair-wise comparisons. All statistics were performed using Systat v12 (Systat Software Inc., San Jose, CA, USA).

## 3. Results

### 3.1. Annual Generations and Seasonal Development

**Figure 2:**
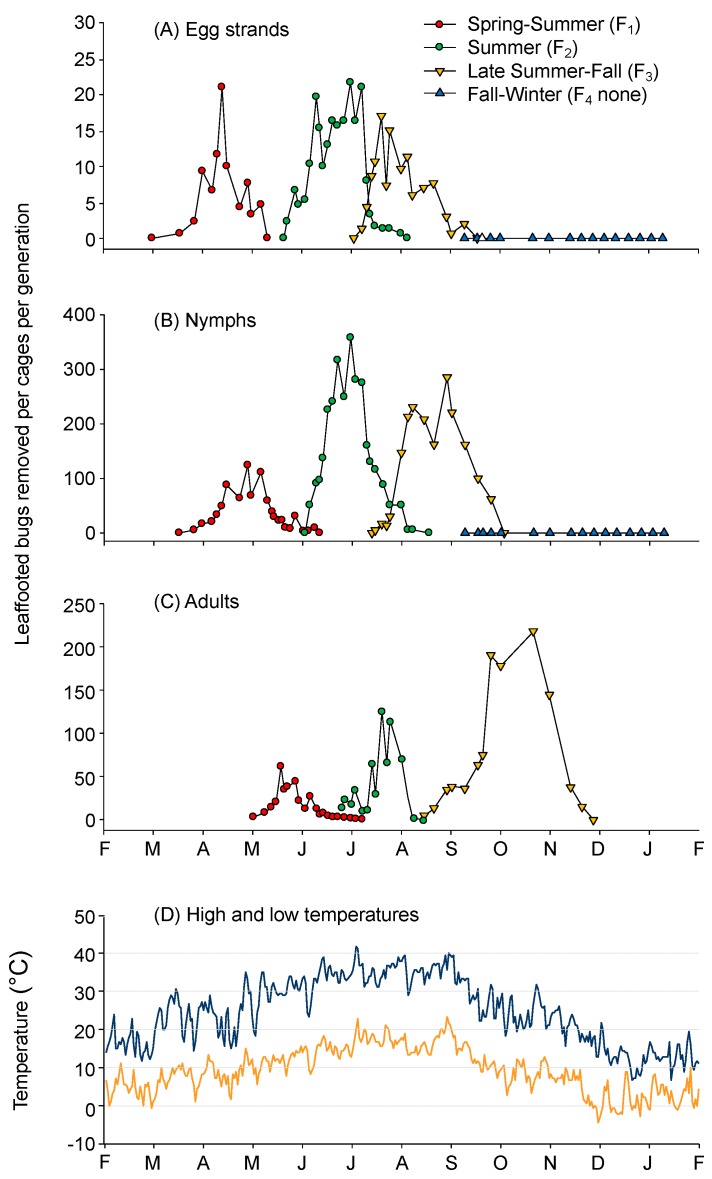
The number of *Leptoglossus zonatus* (**A**) egg strands, (**B**) nymphs, and (**C**) adults recorded every 3–7 days by counting new egg strands and moving and counting nymphs and adults into successive cages; and (**D**) high and low temperatures during the study as recorded by a weather station at the Kearney Agricultural Research and Extension Center, near Parlier, California.

From the initial three cages, each provisioned with 25 adult females from an overwintering population, we recorded a total production of 1214 egg strands, 16,692 nymphs, and 4900 adults over the one-year period. From new egg strands and collected nymphs and adults, a clear picture of the seasonal pattern shows three generations per year under ambient conditions in the San Joaquin Valley (Figure 2).

Overwintered adults were observed mating in the holding cage in late March, and soon after the three F_0_ cages were initialized, mating was observed in all cages (1–7 April). Egg strands from the overwintered adults were first recorded between 18 April (cage 3) and 2 May (cages 1 and 2), with a peak on 14 May and no new egg strands found after 7 June (Figure 2A). There was a prolonged period of egg production in each generation, resulting in some overlap of nymphs and adults from successive generations. New egg strands were recorded from 18 April to 11 June (F_1_), 27 June to 31 August (F_2_), and 7 August to 9 October (F_3_).

The number of nymphs hatching per egg strand was 11.3 ± 2.5, 14.3 ± 1.0, and 12.9 ± 2.3 in the first, second, and third generations, respectively. The sex ratio (F:M) was 0.97:1, 1.01:1, and 1.90:1 in the first, second, and third generations, respectively, although the third generation average was heavily skewed by cage 3, where 1430 adults were collected with a 3.54:1 ratio. Nymph mortality, from 2nd–4th instar, was estimated by the number of nymphs added to each cage minus the number of adults later removed. Using this approach, we found there was a 39.5 ± 10.4, 20.3 ± 5.2, and 43.0 ± 6.0% survival rate of nymphs to the adult stage in the first, second, and third generations, respectively. The number of live vs. dead adults remaining in the cages after egg deposition ceased for overwintering, first and second generations was 14.7 ± 9.3%, 5.2 ± 1.4%, and 44.6 ± 22.0%, respectively, and 91.5 ± 0.8% in the third generation before egg deposition began (these formed the overwintering adult population).

#### Egg Strand Production and Egg Development

Of the adults that produced egg strands (about 60%), overwintered adults (n = 18) produced 5.1 ± 0.9 egg strands that totaled 61.7 ± 11.3 eggs per female. Once isolated, females lived for 38.4 ± 4.3 days with the oviposition period from 2 April to 14 May. Egg development time, from oviposition to 1st instar, was 22.2 ± 1.0 days with 67.1 ± 5.0% successful hatch rate. Adults from the first generation (n = 21) had similar egg strand production and egg development, producing 2.4 ± 0.4 egg strands that totaled 58.8 ± 10.5 eggs per female. First generation adult females lived for 34.5 ± 3.8 days with egg strands deposited from 2–30 July. Egg development time, from oviposition to 1st instar, was 10.9 ± 0.7 days with 86.8 ± 4.3% successful hatch rate. Second generation adult females (n = 18) lived for 34.5 ± 3.8 days with egg strands deposited from 2–30 July. Egg development time, from oviposition to 1st instar, was 10.9 ± 0.7 days with 86.8 ± 4.3% successful hatch rate. Few of the isolated adults (n = 8) in the third generation produced an egg strand, and isolated females lived for only 12.3 ± 1.3 days, producing 1.3 ± 0.3 egg strands that totaled 41.7 eggs per female. Egg development time was 4.6 ± 1.1 days with an 80.2 ± 14.6% successful hatch rate.

### 3.2. Overwintering Survival of Nymphs and Adults

**Figure 3:**
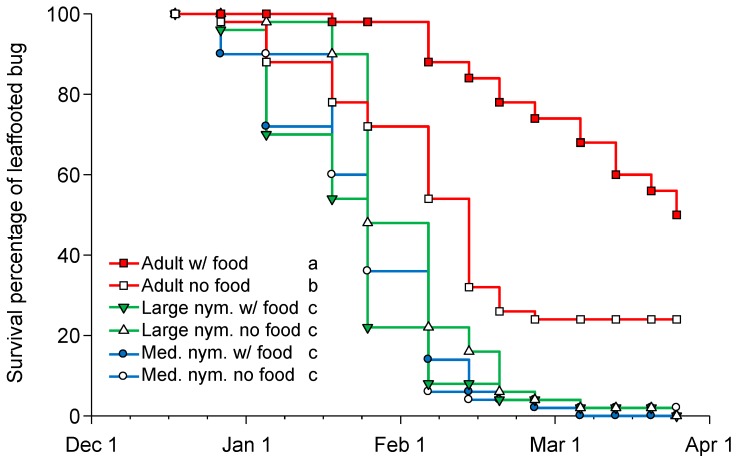
Survivorship curves for medium-sized nymphs (2nd–3rd instars), large-sized nymphs (4th–5th instars), and adult *Leptoglossus zonatus* provisioned with and without food during the winter period (15 December to 26 March) showing adults lived longer than nymphs both with food (Mantel–Haenszel log-rank χ^2^ = 99.23, df = 2, *p* < 0.001) and without food (Mantel–Haenszel log-rank χ^2^ = 26.51, df = 2, *p* < 0.001) provisioning. Different letters following each life-stage and food combination in the key indicate a significant difference in survival analysis pairwise comparisons (α = 0.0033).

There was an impact of life stage on *L. zonatus* overwintering survival (F = 82.415, df = 2,296, *p* < 0.001), but there was no impact of food provisioning (F = 3.411, df = 1, 296, *p* = 0.066). The trial was stopped after 104 days when there were no live nymphs, but there were still 25 and 12 adults alive in the food and no food treatments, respectively; for this reason, the average longevity underestimates adult longevity. During the tested period, adults provisioned with food lived significantly longer (85.6 ± 2.7 days) than adults without food (58.1 ± 3.8 days), and both adult treatments lived longer than any of the nymph treatments (Tukey’s HSD test, *p* < 0.05), which averaged, during the trial period, 40.9 ± 1.7 days and 38.4 ± 1.6 days for large- and medium-sized nymphs, respectively (with and without food treatments combined). Results were similar using survival analysis with adults living longer than nymphs that did not develop to adults or survive the winter period during the 101-day trial (Figure 3).

## 4. Discussion

*Leptoglossus zonatus* has a wide geographic range across its presumptive origins in the Western Hemisphere [27,28] and an equally broad range of crops that it can damage (e.g., [29,30,31,32,33]). Here, we report that in California’s San Joaquin Valley, *L. zonatus* has three distinct generations per year (Figure 2) with the adult stage being the principle overwintering stage (Figure 3). In comparison, other large bug pests of almond and pistachio commonly have slower temperature development, such as *C. hilaris* [34], resulting in two generations per year. Still, the main concern for both leaffooted bugs and stink bugs may not be their seasonal increase in the almond or pistachio orchard but the number of overwintered adults and their proximity to the orchard. *Leptoglossus* species have been reported to move from non-crop habitats into crop systems [35] or between crop systems throughout the season [30]. Therefore, understanding the seasonal population dynamics and the overwintering survival may be vital to developing monitoring and control programs.

There are varying reports on *L. zonatus* developmental and survival rates, depending not just on the populations’ geographic location but on host plant as well [26,32,36]. We found ~60- and ~50-day periods between peak nymph densities from the first to second and second to third generations, respectively (Figure 2B), suggesting summer generations took about 60 days with daily high temperatures ranging between 30 to 40 °C (Figure 2D). From laboratory studies at constant temperatures, *L. zonatus* nymphal development was reported as 24 and 79.5 days at 35 °C and 20 °C, respectively [37]; 21 days on physic nut (*Jatropha curcas* L.) at ~30 °C [36]; 42 days at 30 °C on corn [26]; and 69.1 days at ambient temperatures ranging from 21 to 29 °C on green beans and corn [38]. The reported constant development ranges fit well with our findings on generational periods at ambient temperatures in California’s San Joaquin Valley during the growing season. We also report relatively high nymphal mortality, from 20.3% to 43.0%, based on the number of 2nd–4th instars collected relative to the number of adults produced. These mortality levels are actually lower than those reported by Panizzi [26], where nymphal mortality was 50%, 78.8%, and 85% on corn, soybean, and green beans, respectively. Duarte et al. [38] reported that only 3.7% of *L. zonatus* eggs reached the adult stage and cited research suggesting that high mortality is common when coreids are reared in individual containers. Our recorded mortality, while not measured by development stage, was observed to be predominantly in the 2nd–3rd instars; there was some mortality during the transfer process between cages as well as mortality in cages with high populations where providing fresh food every 3–4 days may not have been adequate to support such populations. We also note mortality in December and January of the third-generation nymphs that did not advance to the adult stage, whereas there was little adult mortality during this period, which was confirmed in the winter survival study.

There are only a few studies of *L. zonatus* adult longevity and fecundity, most of which were completed under controlled environmental conditions and report higher adult longevity and fecundity than we found. Once isolated in the Petri dishes for the egg deposition study, adults produced in the first and second generation lived only 12.3–38.4 days, which was much shorter than expected. For example, the overwintered generation adults were collected on 9 November and held in cages as a group for 140 days before being isolated. We suspect, as suggested by Duarte et al. [38], that individual isolation may have shortened the insects’ lifespan. Depending on the generation tested, isolated adults produced between 41.7–61.7 eggs per female and egg development time ranged from 4.6 to 22.2 days with 67.1–86.8% successful hatch rate. In comparison, Duarte et al. [38] reported *L. zonatus* adult female longevity as 134.5 days with 143 eggs per female. Results from Panizzi [26] are more in line with our study, with female longevity at 43 days, with 5.2 egg strands and 107.6 eggs per female. Both authors report a relatively long pre-oviposition period of 22–36 days. On a meridic diet at constant temperatures, *L. zonatus* females produced 153.0, 347.7, 334.7, and 74.0 eggs per female at 20, 25, 30, and 35 °C, respectively [37]. The higher fecundity reported by these authors may have resulted from the isolation of females in Petri dishes, higher ambient temperatures than the controlled temperatures tested, and our use of green beans as a sole food source. Other researchers have reported a lower fecundity of ~39 eggs per female with a 98% hatch rate [25]. Moreover, our results using isolated females are close to those with collected nymphs per female in our generation study of 32.5 ± 9.6, 21.7 ± 4.5, and 7.3 ± 2.9 in the overwintered, first, and second generations, respectively.

One of the more important findings was that adults are the overwintering stage (Figure 2). In California’s Central Valley, there has been an apparent shift from *L. clypealis* as the primary coreid pest of pistachio and almonds [39] to *L. zonatus* [33]. The shift may be due to a natural expansion of *L. zonatus* range or the increased planting of almond, pistachio, and pomegranates in California. Each of these crops may present suitable host plant material at a different time in the season, with almonds presenting a susceptible crop from February to May, pistachios from April to July, and pomegranates from August to November. Pomegranates in particular provide an excellent fall host where the third generation nymphs can develop to overwintering adults that will then move to sheltered areas from about December to March and then into nut crops from March to May [5]. As mentioned, a broad range of host plants is recorded for *L. zonatus* with variable developmental success [25,26,33,36,37]. Because the overwintering adult population is the principle population damaging the new and most susceptible almonds and pistachio crop [10], a late fall host that supports the development from nymph to overwintered adults may be the key element in the development of large and potentially damaging winter populations. For example, in Alabama, *L. zonatus* adults move from crop fields into nearby fruit orchards in the fall when the fruits start to ripen [30]. A similar situation could take place in California, where large populations that develop on pomegranates in the fall leads to large overwintering adult populations that then damage nearby almond and pistachio orchards the following spring. Still to be determined is whether pomegranates or other ‘super food’ sources might support a fourth generation in the San Joaquin Valley, leading to large overwintering populations.

## 5. Conclusions

California’s San Joaquin Valley is one of the world’s most important fruit and nut production regions; the coreid *L. zonatus* has recently emerged as the primary hemipteran pest of the multi-billion-dollar (US) almond and pistachio industries in this region. Currently, there are no commercially available tools to effectively monitor populations that migrate from their overwintering sites into commercial nut crops, and here we sought to improve our understanding of this pest’s seasonal dynamics and abundance. We report that *L. zonatus* has three generations per year, with adults being the principle overwintering developmental stage. Compared with previous studies, adult *L. zonatus* reproductive potential and the resulting seasonal increase in population abundance was not as high as reported in the southeastern USA and South American studies, and we suspect our resident population may be dampened by high summer temperatures and poor food sources. The ability of the third and final generation to locate a suitable summer–fall host for nymphal development to the overwintering adult stage may be a key element in developing successful control programs. Better monitoring of overwintered adults migrating into nut crops is needed, as well as a better understanding of the role pomegranates play in sustaining late fall populations.
